# Successful treatment of life-threatening mycobacteriosis using adjunctive gamma-interferon therapy with genetic analysis

**DOI:** 10.5588/ijtldopen.23.0406

**Published:** 2024-01-01

**Authors:** P. Confalonieri, S. Maiocchi, F. Salton, B. Ruaro, C. Rizzardi, M. C. Volpe, D. Licastro, L. Braga, M. Confalonieri

**Affiliations:** ^1^ Pulmonology Department, University of Trieste, Trieste,; ^2^ International Centre for Genetic Engineering and Biotechnology, Functional Cell Biology, Trieste,; ^3^ Department of Life Sciences, University of Trieste, Trieste,; ^4^ Pathology Unit, University of Trieste, Trieste,; ^5^ Genomics Lab, Area Science Park, Trieste, Italy.

**Keywords:** IFN-γ, mycobacterial infections, tuberculosis, NTM, non-tuberculosis mycobacteria

Dear Editor,

Both TB and non-tuberculosis mycobacteria (NTM) can lead to life-threatening disseminated diseases, which often defy conventional treatments, especially in individuals harbouring multidrug-resistant (MDR) bacilli or in those with altered immune responses.^1–4^ A previous study, involving six HIV-negative patients with refractory disseminated NTM infection, revealed promising outcomes following subcutaneous administration of recombinant interferon-gamma (IFN-γ) over 8 weeks.^5^ IFN-γ plays a pivotal role in acquired immune responses against pathogenic mycobacteria and intracellular pathogens, suggesting its potential as an adjunct to antibiotics in combating persistent mycobacterial infections.^6^ However, the efficacy of IFN-γ as an adjunctive therapy in non-severe cases has yielded inconsistent results.^7,8^

In 2008, we first explored the potential of IFN-γ rescue therapy in conjunction with antimycobacterial drugs. We used subcutaneous IFN-γ in a critically ill young female with HIV-negative puerpera who, following splenectomy due to splenic rupture from disseminated *Mycobacterium avium* complex (MAC) infection, remained unresponsive to standard anti-MAC therapy after 40 days. Adjunctive recombinant IFN-γ (IMUKIN gamma-interferon-1b recombinant human 2 million IU/0.5 mL injection vial; Boehringer Ingelheim International, Ingelheim, Germany) subcutaneously at 100 mcg three times per week was administered until complete eradication of the mycobacterial infection and patient recovery. Local ethical approval was obtained (ethics committee from A.O. Universitaria Ospedali Riuniti di Trieste, #AOTS-Dec-2008). Encouraged by this clinical breakthrough, we employed the same protocol in five more cases of life-threatening disseminated refractory TB or NTM infections over the next 14 years at the Pulmonology Department of the University Hospital of Cattinara, Trieste (a designated centre for rare diseases within the European Reference Network-Lung). We collected and stored whole blood samples from the six patients for subsequent genetic analysis using next-generation sequencing (NGS; Illumina’s NovaSeq6000 platform, Albany, NY, USA). All patients provided informed consent to both genetic analysis and IFN-γ treatment. The key clinical and genetic attributes of patients treated between December 2008 and September 2022 are shown in the Table. All patients had a confirmed diagnosis of mycobacterial infection on culture with drug susceptibility testing. Among them, two had *M. tuberculosis* hominis, one drug-susceptible and one MDR-TB, whereas the remaining four had drug-susceptible NTM infections.

**Table.** Clinical and genetic characteristics of the patients.^†^

Characteristic	Patient #1	Patient #2	Patient #3	Patient #4	Patient #5	Patient #6
Sex	Female	Male	Male	Male	Male	Female
Age at admission, years	31	47	69	44	71	75
BMI, kg/m^2^	20	23	25	26	17	15
Isolated mycobacterium	MAC	*M. tuberculosis hominis* (QuantiFERON-negative)	MAC	MAC	*M. tuberculosis hominis* (QuantiFERON-negative)	*M. chimaera*
Infection localisation	Lungs, lymph nodes liver, spleen, skin	Miliary pulmonary TB	Lungs, mediastinal lymph nodes, vertebrae	Lungs, mediastinal lymph nodes, brain	Miliary pulmonary TB, bones, brain, pleura	Lungs, throacic lymph nodes
Respiratory support	O_2_ + NIV	Invasive mechanical ventilation, ETI	O_2_ + NIV	O_2_	Invasive mechanical ventilation, ETI	HFNC + NIV
Antibiotic therapy	EMB + AZM + RBT	RIF + INH + PZA + EMB + AMK	EMB + clarithromycin + RBT	EMB + AZM + RBT + AMK	INH + RIF + PZA + AMK, after 3 months EMB, levofloxacin, protionamide, clofazimine	RIF + AZM + AMK + linezolid
Complications during anti-mycobacterial treatment	Rupture of the spleen (surgically removed)	Severe acute respiratory failure, ARDS	Spleen rupture, spondylodiscitis	Respiratory failure	Severe sepsis, septic shock, pancytopenia	Severe respiratory failure, sepsis, MOFS, Pulmonary infection by MDR *Klebsiella Pneumoniae* + *Candida alb*.
Comorbidity	Sarcoidosis, severe idiopathic B lymphopenia	Sarcoidosis	Valvular cardiopathy, mild chronic renal failure	COPD, thrombocytopenia, HBV + chronic hepatitis, β-thalassemia minor	Sarcoidosis	Non-cystic fibrosis severe bronchiectasis, COPD
Deficient lymphocyte sub-population	CD19+ 0.5 cells/μL CD4+ 238 cells/μL	CD4+ 86 cells/μL, CD8+ 120 cells/μL	None	CD4+ 39 cell/μL CD8+ 31 cell/μL	CD19+ 14 cell/μL CD3+ cells 159 cell/μL CD4+ 127 cell/μL CD8+ 28 cell/μL	None
Blood CD4/CD8 ratio (normal value: 1.5–3.5)	0.4	0.72	0.5	1.25	4.58	1.2
Genetic abnormalities^‡^	CCL4L2 homozygous for C221G (NM_001291471) FCGBP homozygous for A3488G*, G3489C*, C3494A, C3521A, T3530C, T4270C, T4541C, (NM_003890) MUC5AC homozygous for C738G, T9139A, A9700G, T13379C, C13382T (NM_001304359)	CCL4L2 homozygous for C221G (NM_001291471) FCGBP homozygous for A3488G*, G3489C*, C3494A, C3521A, T3530C, (NM_003890) MUC5AC homozygous for C738G, T9139A, A9700G, T13379C, C13382T (NM_001304359)	CCL4L2 homozygous for C221G (NM_001291471) FCGBP homozygous for A3488G*, G3489C*, C3494A, C3521A, T3530C, T4270C, T4541C (NM_003890) MUC5AC homozygous for C738G, T9139A, A9700G, T13379C, C13382T (NM_001304359)	CCL4L2 homozygous for C221G (NM_001291471) FCGBP homozygous for A3488G*, G3489C*, C3494A, C3521A, T3530C, T4270C, T4541C (NM_003890) MUC5AC homozygous for C738G, T9139A, A9700G, T13379C, C13382T (NM_001304359)	CCL4L2 heterozygous for C221G (NM_001291471) FCGBP homozygous for A3488G*, G3489C*, C3494A, C3521A, T3530C, T4270C, T4541C (NM_003890) MUC5AC homozygous for C738G, T9139A, A9700G, T13379C, C13382T (NM_001304359)	FCGBP homozygous for A3488G*, G3489C*, C3494A, C3521A, T3530C, T4270C, T4541C (NM_003890) MUC5AC homozygous for C738G, T9139A, A9700G, T13379C, C13382T (NM_001304359)
Duration of IFN-γ treatment, months	12	7	12	9	4, until death	3, until death
Outcome	Mycobacteria eradication; subsequent pulmonary opportunistic infections some years later	Mycobacteria eradication	Mycobacteria eradication	Mycobacteria eradication	Non-mycobacteria eradication; death after 4 months of TB treatment	Non-mycobacteria eradication; co-infection of *Candida* sp. complicated by severe sepsis and MOFS; death after 3 months of NTM therapy

† Levels of lymphocyte sub-population was referred to at time of diagnosis before starting of IFN-γ therapy. All genetic mutations were non-synonymous SNV type. The gene nucleotide variants and corresponding protein variants are as follows: *CCL4L2* (reference transcript NM_001291471) C221G and P74R; *FCGBP* (reference transcript NM_003890) A3488G*/G3489C*/C3494A/C3521A/T3530C/T4270C/T4541C and, respectively, E1163G*/E1163D*/A1165D/A1174D/V1177A/S1424P/V1514A; MUC5AC (reference transcript NM_001304359) C738G/T9139A/A9700G/T13379C/C13382T and, respectively, D246E/S3047T/I3234V/L4460P/P4461L.

‡ Regarding *FCGBP,* the two SNVs occur in the same codon, therefore resulting in the substitution of glutamate with glycine.

BMI = body mass index; MAC = *M. avium* complex; O_2_ = oxygen; NIV = non-invasive mechanical ventilation; ETI = endo-tracheal intubation; HFNC = high-flow nasal cannula oxygen; EMB = ethambutol; AZM = azithromycin; RBT = rifabutin; INH = isoniazid; PZA = pyrazinamide; AMK = amikacin; RIF = rifampicin; ARDS = acute respiratory distress syndrome; MOFS = multi-organ failure syndrome; SNV = single- nucleotide variation; MDR = multidrug-resistant; COPD = chronic obstructive pulmonary disease; HBV = hepatitis B virus; IFN-γ = interferon-gamma; NTM = nontuberculous mycobacteria.

At the point of diagnosis, four of the six patients exhibited varying degrees of lymphocytopenia, either with complete type-B lymphocyte suppression, general leucopenia or T-cell lymphopenia. All the patients had diffuse pulmonary involvement with respiratory failure requiring several types of respiratory support (e.g., non-invasive ventilation, invasive mechanical ventilation, or oxygen). Following initial treatment with three to six antimycobacterial drugs (and after 1–2 months of unresponsive therapy and progression of critical illness), subcutaneous injections of IFN-γ were introduced three times weekly. Of the six refractory mycobacteriosis cases, four experienced complete eradication of mycobacteria. Regrettably, only these four survived. One patient had to discontinue IFN-γ prematurely due to aggravated pancytopenia. However, IFN-γ treatment proved safe in most patients, with minimal side effects apart from manageable fever (resolved using paracetamol).

Genomic DNA was extracted from peripheral whole blood and subjected to whole exome sequencing using NGS Illumina technology. We aimed to identify potential genetic factors underlying susceptibility to refractory TB and NTM, and favourable response to IFN-γ treatment. Given the limited cohort size, we adopted a population frequency cut-off of <0.00036, determined by applying the Hardy-Weinberg formula with assumptions regarding the frequency of severe mycobacteriosis and the phenotypic relevance of homozygous mutations. This process identified 52 genes carrying homozygous, non-synonymous single-nucleotide variant, and exceptionally rare genetic alterations. To assess the biological significance of each mutated gene in immune response, monocytes from peripheral blood of three patients and three healthy controls were isolated. These were differentiated into monocyte-derived macrophages (MDMs) following 8 days incubation in RMPI-1640 with 10% normal human serum to allow their natural transition from monocytes to resting macrophages. Subsequently, resting macrophages were stimulated with lipopolysaccharides (LPS) for activation. Post-treatment, MDMs were harvested for bulk messenger RNA (mRNA) sequencing using NGS Illumina technology. The genes that were expressed by MDMs (pBasemean >10), and possibly, differentially expressed after LPS treatment were considered as essential during native immune response to LPS, as a surrogate scenario of mycobacteria exposition. Among the 52 commonly altered genes, only 19 were expressed in patient-derived macrophages. In particular, the *CCL4L2* (C-C motif chemokine ligand 4 like 2) gene demonstrated a noteworthy increase in mRNA expression in LPS-stimulated patient MDMs compared to healthy MDMs. This increase in *CCL4L2* mRNA in LPS-stimulated patient MDMs (4.16 log2FC vs. unstimulated patients’ monocytes) was 2.47 times higher than the increase in LPS-stimulated healthy MDMs (1.68 log2FC vs. unstimulated healthy monocytes). Intriguingly, *CCL4L2* was found to be homozygously mutated in all four IFN-γ responder patients and heterozygously mutated in one of the non-responders. The *CCL4L2* gene encodes for CCL4L2, a chemokine that induces chemotaxis of cells expressing CCR5 (C-C chemokine receptor type 5), and it is one of several cytokine genes that are clustered on the q-arm of chromosome 17. CCL4L2, which is a paralog of the *CCL4* (C-C motif chemokine ligand 4) gene, is associated with immune cell migration via the CCR5 chemokine receptor found in diverse immune cells (macrophages, immature dendritic cells, granulocytes and T-cells),^9^ thus playing a key role in TB susceptibility control^10^ and progression to active TB.^11^ Furthermore, *CCL4L2* is vital in mediating macrophage response to cytosolic DNA sensing, a pathway activated during mycobacterial infection.^12^ Similarly, FC gamma binding protein gene (*FCGBP*), which carried five homozygous non-synonymous mutations in all six patients and two additional homozygous non-synonymous mutations in five out of six, exhibited downregulation in unstimulated patient MDMs compared to healthy MDMs. This pattern persisted after LPS stimulation, suggesting reduced gene expression in the patient’s immune response. *FCGBP*, a mucin-like protein binding immunoglobulin G, contributes to mucosal immunity^13^ and discriminates between active and latent TB.^14^ Likewise, the MUC5AC gene with three homozygous non-synonymous mutations in all six patients and two more in five out of six patients, displayed high expression in upper and mid airways Club cells. A linked polymorphism, rs28737416, was correlated with disease severity in *M. tuberculosis* infection,^15^ highlighting its relevance in immune response activation upon mycobacterium exposure.

In conclusion, our case series of patients with severe disseminated mycobacterial infections showed that adjunctive IFN-γ treatment proved effective in four out of six cases. We identified two genes potentially correlating with NTM and TB severity (*MUC5AC* and *FCGBP*), and one (*CCL4L2*) suggesting a positive response to IFN-γ therapy. Further research is required to validate these findings. We propose the extended use of INF-γ as a rescue therapy in non-responder-severe cases of mycobacteriosis and encourage the use of genetic analysis to predict outcome and response to INF-γ treatment.
